# HURRICANE STRESSORS AND BUFFERS IN RELATION TO DEPRESSIVE SYMPTOMS AMONG OLDER ADULTS IN PUERTO RICO

**DOI:** 10.1093/geroni/igad104.0848

**Published:** 2023-12-21

**Authors:** Victoria Guinn, Sheila Thompson, Erin Ballard, Michael Crowe, Lisa Brown

**Affiliations:** Palo Alto University, Palo Alto, California, United States; Palo Alto University, Palo Alto, California, United States; University of Alabama at Birmingham, Birmingham, Alabama, United States; University of Alabama-Birmingham, Birmingham, Alabama, United States; Palo Alto University, Palo Alto, California, United States

## Abstract

The present study examines the role of religiosity and social support on depressive symptoms and the moderating role of hurricane stress related to friends and family following Hurricane Maria in a sample of Puerto Rican older adults. Data was collected from the Puerto Rican Elder: Health Conditions (PREHCO) study. The present study consists of 613 participants in Wave 3 of the longitudinal study. Depressive symptoms were measured using the Geriatric Depression Scale (GDS). Social support was measured using the Lubben Social Support Network scale. Religiosity and hurricane stress related to friends and family were measured using survey items developed by PRECHO investigators. Religion items include church attendance and the helpfulness of religion for health conditions. A linear regression was conducted. Controlling for education and days since the hurricane, church attendance (b=0.642, p<.001), and social support (b=-0.098, p<.001) predicted depressive symptoms. When hurricane stressors related to family and friends were added to the model, church attendance (b=629, p<.001) and social support (b=-0.098, p<.001) remained significant predictors, and hurricane stressors predicted depressive symptoms (b=-0.378, p=.018). Helpfulness of religion was not a significant predictor of hurricane stress. Church attendance and social support are important coping strategies for Puerto Rican older adults. Increased social support and frequent church attendance buffer the development of depressive symptoms even when hurricane-specific stress is present. Future research may examine if church attendance and social support buffer the development of depressive symptoms when other non-social hurricane stressors are present.

